# The Hospital Nursing Supplement

**Published:** 1895-08-17

**Authors:** 


### "The Hospital" Nursing Mirror.

Being the Extra Nursing Supplement of "The Hospital" Newspaper.

[Contributions for this Supplement should be addressed to the Editor, The Hospital, 428, Strand, London, W.C., and should have the word "Nursing" plainly written in left-hand top corner of the envelope.]

### News from the Nursing World.

### IN AID OF A HOSPITAL.

The bazaar opened last week at Banff in aid of the Chalmers' hospital, by the Duke and Duchess of Fife, was well attended. The stalls, which were particularly attractive, were visited by the Duke and Duchess, who made many purchases before inspecting the guard of honour of the Banff Volunteer Artillery, of which the Duke of Fife is honorary colonel.

### A WARNING TO NURSES.

Last week a nurse applied at the Marylebone Police Court for advice respecting a registry office, by which (as the magistrate himself said) persons all over London have been swindled. According to the press report the nurse applied to an office in the Edgware Road, and the proprietress undertook, on her paying half-a-crown, to get her an engagement. Two addresses were subsequently supplied, but neither of the ladies named required a nurse, the servants at the second house stating that nurses were continually sent there from the registry office, although no one was required. The magistrate, Mr. Hannay, is reported to have promised to do all possible to assist the nurse if she would get further information, and apply again to him. We trust the publicity thus given to these proceedings will act as a salutary warning to nurses coming to London.

### CAN THIS BE TRUE?

"You have made a mistake," said a private nurse, handing back the cheque as she spoke. Her employer looked puzzled. "Do you think it isn't enough, nurse?" she inquired. "It is just what I have given before." "I don't understand," said nurse. "I came to you for two guineas a week, exactly a month ago, and you want to give me thirteen guineas. Will you alter the cheque, or shall I give you the change?" "Neither," was the impatient answer. "I am willing to give you a present of three guineas, as I have done on previous occasions when I've had a professional nurse; but I really cannot offer any other form of gift, for I am not able to go and choose a present for you." Nurse, however, proved an exception to her predecessors. "Thank you," she said firmly, "but I shall accept nothing beyond my just fees, and I think that those who do so, except in most exceptional circumstances, disgrace the whole nursing profession!" The employer smiled. "You must be new to private nursing, I think," she remarked, "or you would know that most people are expected to give an honorarium; only, of course, the recipients don't talk about it."

### MACCLESFIELD INFIRMARY.

The *Macclesfield Courier* seems to be under the impression, with many people in Macclesfield, that a hospital does not need a matron to look after its nursing staff, and that they would be likely to flourish better without than with the supervision of such an officer. This is, of course, an original view to take of an important question affecting hospital administration. It would not, however, be entitled to serious consideration if the public proceedings of the governors and committee of the Macclesfield Infirmary in the past had afforded reasonable evidence of sound knowledge of hospital administration. Seeing, however, that the proceedings in question are a grave scandal, in that they tend to disorder and confusion in the management of the hospital, we incline to the view that unless some wisdom and moderation are displayed by the committee, it may be necessary to close the institution altogether in the best interests of the community which it was established to serve. No doubt there are earnest and good men resident in the town of Macclesfield who have their eye upon the proceedings referred to. We hope, and believe, that wiser counsels may prevail, and that an effort will now be made to secure sound discipline and administration at the infirmary under the new matron, Miss Bowman, who will, we trust, be supported in the discharge of the very difficult position she has to face. Any attempt to abolish the office of matron should be met by an entire change in the *personelle* of the committee of management, and the introduction of such radical reforms in the administration as will secure not only peace but efficiency at the Macclesfield Infirmary.

### AN IRISH WORKHOUSE.

The report of the medical officer at Nenagh Workhouse, as given in the *Freeman's Journal*, shows a deplorable absence of nursing in that institution. He asserted that patients got out of bed in the night and died on the floor, where they lay for hours, in the absence of any night nurses. One of the Guardians is reported to have said, "It is horrible to think of the treatment to which these infirmary patients have been subjected." The committee of Guardians appointed to inquire into the nursing arrangements strongly favoured the appointment of a paid night nurse, "to prevent the continuance of the scandalous state of things," but the suggestion was actually over-ruled by the majority of Guardians, who decided to try pauper inmates on night duty for a month.

### BRADFORD MEDALS.

The annual distribution of medals at Bradford Infirmary was well attended, the Mayor presiding on the occasion. The gold medal was awarded to Nurse M. H. Paterson, who gained the highest number of marks in the recent examinations. Nurses A. M. Hamer and E. Walton were bracketted for the second prize, therefore the silver medal will remain for half the year in the possession of each of these nurses in turn. Handsomely bound volumes of poetry were given to all the three nurses by Mr. W. C. Lupton, chairman of the House Committee.

### NURSING IN ABERDEEN.

A bazaar will take place in October for the purpose of raising funds for the Aberdeen District Nursing Association. An excellent house, which has been adapted for a nurses' home, requires furnishing, and the increasing work of the association makes great demands on the funds, and therefore liberal support is desired and deserved. The association, which is affiliated with the Scotch branch of the Queen's Jubilee Institute, supplies fully-trained nurses to the sick poor. Starting with a single nurse in 1892, the staff now consists of a superintendent, three nurses, and a Queen's probationer. It is to be hoped that the interest already shown in the autumn bazaar will result in a handsome addition to the finances of this excellent charity.

### CAN SIX MONTHS' SUFFICE?

The announcement that the Sutherland Benefit Nursing Association "aims at providing one or more nurses for each of the thirteen parishes in the county" would be worthy of unqualified commendation if the scheme were consistently carried out. The recently-issued rules of the association, however, state that the so-called "nurses" are country women, who have undergone six months' training only. Surely the influential committee of this well-intentioned scheme would be more likely to benefit the sick poor by supplying a smaller number of better qualified women. Already in many parts of Scotland the Queen's Nurses are established by means of the branch of which the Princess Louise is president. These intelligent, educated women have to undergo a full term of training both in hospital and district nursing before their names are placed upon the roll. It is obvious that cottagers, however capable and willing, must require at least an equal amount of instruction to entitle them to be called nurses.

### A SUCCESSFUL CEREMONY.

The fine memorial hospital at Crewe was formally opened on the 7th inst. by the Earl of Crewe, in the presence of a large and distinguished company. After the handsome golden key, presented by the chairman, Mr. Webb, had been duly used, the hospital was thoroughly inspected by Lord Crewe, the Bishop of Chester, the Hon. R. Ward, Mr. and Mrs. Yates Thompson), Lord Stalbridge (chairman of the London and North-Western Railway Company), Lady Fitzgerald, and others. The dedicatory prayer was said by the Bishop, and a hymn sung by the Crewe Philharmonic Society, after which excellent speeches were made by the Bishop, Lord Crewe, Lord Stalbridge, Mr. Webb, the Mayor, and the Hon. P. Ward. Another hymn and the National Anthem sung by the Philharmonic Society concluded a most successful meeting. The hospital was then inspected by many hundreds of people, and as it was impossible for all who assembled to obtain admittance, the new building was again opened to the public on the following day, and the first patients were received on the 12th instant.

### WORK IN HOLLAND.

From the eleventh report of the Protestant Deaconesses' Institute, Arnhem, we learn that Her Majesty the Queen Regent has been graciously pleased to become a patroness of the institute. The completion of the new buildings is a notable improvement by which the hospital accommodation will be greatly increased. The work of the deaconesses both in and outside the hospital has continued uninterrupted, the number of in-patients received during the past year having reached the total of 691.—The Children's Hospital at Utrecht, in its seventh annual report, makes an earnest appeal for increased funds, without which it is quite impossible that the institution can continue to carry on the excellent work it is doing, and it may, in fact, have to be erased from the list of the philanthropic institutions at Utrecht. During the past year 111 children have been treated free, the expenses of 52 others being paid either by parents or other relatives or friends.

### OUR AMERICAN SISTERS.

Outdoor uniform, hitherto little favoured by our American sisters, appears to be gaining popularity just now. It is occasionally to be seen in the New York streets, and is distinctive of the Erie County Hospital staff. This institution is adopting grey dresses, cloaks, and bonnets for the street wear of their nursing staff, and pink and white for indoor uniform. The superintendent of nurses, Miss Lowe, received her training in England and has also a large experience of New York hospitals.

### PROGRESS AT BALTIMORE.

The opening of a training school for nurses in Baltimore in connection with the Royal Victoria Hospital has been followed by a year's successful work. This fine new hospital has accommodation for 260 patients, but three wards, containing in all 80 beds, are not as yet opened. In the first year 350 applications were received for admission to the training school, and the staff now consists of a lady superintendent, a housekeeper, night superintendent, 10 graduates, 26 pupils, and three probationers. Lectures on physiology are given by Drs. Adami and Martin, on hygiene by Dr. Craik, on diseases by Dr. Stewart. A library has been formed, and Miss Edith Draper, superintendent of the nurses, strongly advocates the establishment of a diet kitchen, in which the nurses could be practically instructed in sick cooking.

### WOMEN'S WORK IN INDIA.

Very interesting accounts of medical and mission work in India have been given by Miss Haskew, M.D., whilst on furlough in England. For two and a-half years she has had sole charge of the Lady Kinnaird Memorial Hospital at Lucknow, and has, therefore, many personal reminiscences of work amongst the native women of India.

### SHORT ITEMS.

Sister Henrietta, formerly head of the nursing department at Kimberley Hospital, has now a staff of private nurses working under her at S. Michael's Home.—The "Trained Nurses' Club," 12, Buckingham Street, Strand, will be closed from August 19th to September 2nd, but letters marked, "to be forwarded," will receive immediate attention.—The Newport Isle of Wight District Nursing Society has issued its sixth annual report, which shows a small balance in hand, and a large record of cases visited.—The report of the Children's Home, Much Hadham, shows that 105 children of ages varying from a few months to thirteen years have been cared for in the course of the last twelve months with excellent results.

### Elementary Physiology for Nurses.

By C. F. Marshall, M.D., B.Sc., F.R.C.S.

### II.—INTRODUCTION (*continued*).

### The Respiratory System.

Suppose we have a steam-engine, in which all parts are properly made and in good order; the boiler filled with water, and a good supply of fuel. Yet our engine will not start; something more is necessary, we must light the fires, to convert the water into steam in order to move the piston. So it is with a clock or watch; it must be set going.

So also with an animal; it must be set going and kept going, and the mode in this case is more like that of an engine than a clock, for it is by burning that the necessary energy is supplied to set an animal going. This burning is a chemical process carried on by the oxygen gas obtained from the air. A constant supply of this gas is required, for if a furnace is closed so that no air can get at it, the fire will go out; so will an animal die when deprived of oxygen. The wasting of the tissues of an animal is of the same nature as the burning of fuel, viz., a process of oxidation, by which the more complex constituents of the body are reduced to simpler ones, one of the chief of which is carbonic acid gas. As a result of this burning, energy is given out as heat and as muscular action. Hence there are two processes continually going on, a destructive process, called katabolic, and a regenerative, or anabolic process. In order to effect this the animal must be alive.

The steam-engine is set going by the oxygen of the air, so also is an animal, and the respiratory system is the agent which keeps the animal going. Respiration consists of two events—(1) inspiration, the taking in of oxygen gas; (2) expiration, the getting rid of carbonic acid gas. The burning of the animal body is less energetic than in a steam-engine, for there is no evolution of light; but there is considerable evolution of heat, for everyone knows that the body is warm, and that warmth is kept up by the body itself. Clothes do not keep the cold out, but keep the heat of the body in. A bed is warmed a few minutes after we have been in it by the heat of the body.

As it is the burning of the coal in a locomotive which really makes the wheels turn round, so also it is a similar process which, in ourselves, enables us to move, speak, and think. Just as with a certain amount of coal we can get a certain amount of work out of our engine, and if we want more we must stoke up; so with a man, the amount of work he can do depends directly on the activity of the process of burning going on inside him.

### The Excretory System.

As a result of the burning in our engine, there is a certain amount of ash formed by the burning of the fuel. This ash is not only useless, but actually injurious, and if allowed to accumulate will check the fire or even prevent it burning at all. Similarly in ourselves, as a result of the chemical processes which are constantly going on, and by virtue of which we are enabled to do work, certain bodies which we may compare to ashes are formed which must be got rid of. This is effected by the excretory organs. These substances are really harmful, yet they are constantly manufactured within the body itself; in fact, the body is continually trying to poison itself. Excretion is the beneficial process by which this suicidal tendency is kept in check.

Excretory products are chiefly carbonic acid gas and solid matters, of which urea is the most important. The former is got rid of by the lungs, the latter by the kidneys. We saw at the beginning that a man is constantly losing weight, and we see now why this is and what the loss consists of. A man or animal loses weight for the same reason that a locomotive loses weight on a journey—because in order to keep him going there must be a constant supply of heat, obtained by burning; in the case of the engine, of the fuel; in the case of man, of the substance of the body itself. In both cases alike excretory products are formed as a direct result of the activity of the engine or the man; in both cases these are injurious and must be got rid of. In the engine it is chiefly carbonic acid and ashes; in the man, carbonic acid and urea. In both cases water is necessary; in the engine this passes out as it entered, practically the same in amount; in man it is partly formed in the body, but chiefly taken in with the food.

These excretory products are first poured out into the blood, and afterwards withdrawn from it by the excretory organs. The blood therefore performs three functions: (1) To supply food to all parts of the body, (2) to carry oxygen to the tissues, and (3) to carry excretory products away from them.

### The Nervous System.

This is the co-ordinating mechanism by which all the different and mutually dependent parts of the body are brought into communication, and made to work in harmony with one another. If one part of the body is working hard, it must have increased blood supply for the time; both for the supply of food and oxygen, and for removal of waste products. Hence the evil of violent exercise directly after a meal. Again, muscles must not contract spontaneously, but when we wish them to, *i.e.*, we—that is our brains—must be in direct connection with our muscles. This is effected by the nerves.

The nervous system consists of two parts: (1) The central, consisting of the brain and spinal cord; and (2) peripheral, the nerves. We may compare it to a telegraph office. Messages are received from all parts to the central office—the brain. The brain can put any two messages into communication with one another. A message may pass through the central office without any one in the office knowing anything about it, *i.e.*, without involving the will. For instance, if our hand is put down on the sharp point of a pin, it is drawn back, but the message is sent without involving the will.

In the following lectures we shall take the several systems one by one, and consider them more in detail.

### Our Princess's Letter.

Inquiries have been made by our correspondents regarding the letter addressed by the Princess of Wales to the Royal Pension Fund for Nurses after the presentation of a screen by them to Her Royal Highness. We are pleased to state that a few copies of The Hospital of December 13th, 1890, which contained the fac simile of our Princess's kind letter can be obtained at the office, 428, Strand, at threepence each.

### "The Hospital" Convalescent Fund.

### THE NURSES' BED.

The nurse who required a change of air after an attack of pneumonia is deriving great benefit from her stay at Littlehampton. She writes: "I am feeling so much better and stronger, and getting quite sunburnt. It is so restful and peaceful here; it is a delightful place, and the sister is so very kind. I should like The Hospital readers to know what a delightful fortnight they have given me." Subscriptions to the fund should be addressed to the Hon. Secretaries at The Hospital Office, 428, Strand. A donation of 20s. from an anonymous friend at Cambridge is acknowledged with thanks.

### Concerning Nurses.

(By One of Their Friends.)

The general run of handbooks have rather a tendency to speak of a nurse as a sublime kind of creature, hardly belonging to the race of ordinary women, and quite above all weaknesses. Like many another "ideal," this ministering angel does not appeal to our sympathies half so much as the real nurse with whom sickness, sooner or later, brings each of us into contact.

The best sort of nurse is merely the best sort of woman, the most unselfish, patient, gentle, cheerful, and observant of her sex; one, in fact, who would shine in any line of life.

It is all nonsense to string together a long list of virtues and say that in one "trained nurse" all these qualities should be found. On the other hand, a probationer's life in a good school of nursing will do much towards developing the latent good in her. The regular, wholesome life brings out qualities of whose previous existence she herself was probably ignorant. Perhaps some will smile at the word "wholesome" or "healthy" being applied to hospital life. Yet a woman of twenty-five, of sound constitution, and tastes in harmony with her chosen work, if she enters on it voluntarily, with a "God-speed" from her parents, is little likely to fail. An honest desire to avoid giving trouble to others will help her to guard against "breaking down," and she will endeavour to preserve her good health by proper sleep and exercise and regular meals.

Much discussion has taken place respecting nurses' food, and assuredly the nourishment provided for doctors and nurses breathing "hospital air" and living at high pressure should be of the best, both as to cooking and quality. This is quite distinct from the "luxurious living" which no wise person would wish introduced into charitable institutions.

In the handbooks on nursing the ideal nurse has a great many precepts laid down for her behaviour towards her patients, but there appears to be no literature dealing with the treatment of nurses by patients and in hospital wards, of course, there is no difficulty in this matter.

The inmates are not only obedient to the nurses, but they are frequently most considerate for them. Nowhere is an experienced woman so thoroughly appreciated as by these poor folks, who being "workers" themselves are good judges of "skilled labour," and very critical over slovenly performances. A manual for the employers of the private nurse might be compiled with advantage, for although in most cases the nurse carries with her a copy of the rules framed by her own institution for her protection, this is frequently treated as waste paper. For example, the friends of a patient sometimes express surprise when the nurse objects to sleeping in the patient's room, failing to see that in cases of cancer or phthisis this custom would be as undesirable as with diphtheria. Again, it is usual for a nurse to find, when suddenly summoned to a patient in an afternoon, that she is wanted for night duty, the relations thinking that they can "manage in the day" themselves. The hours pass on, and it is not till late in the following afternoon that the weary woman finds herself released, to take a few hours' precarious rest, with the certainty that if the least difficulty occurs, she will be unceremoniously aroused at any moment. A person who sits up regularly at nights must be properly fed, the order of day and night being to a certain extent reversed, and suitable provisions should be always placed conveniently outside the sick-room; but this is very seldom realised in a private household—a nurse being considered rather a glutton if she is not satisfied with a cup of tea and bread and butter. Of course, one night's nursing can be done on this diet, but a professional nurse, who aspires to live long and work well, requires more substantial food.

Servants are generally very kind and considerate to the private nurse, when once convinced that she neither intends to give needless trouble nor yet to "keep out" other people from the sick-room, if their presence does no harm to the patient. One excellent nurse whom we know has a habit of saying to the nearest relative of a new patient, "It must be very hard for you to give up to a stranger. I don't in the least mind your staying all night in the room if you wish it. Or, if you feel tired and will lie down I can call you whenever you like, and then you can see how we are getting on."

Of course there are many women in the ranks of the profession who ought never to have entered it, such, for instance, as the one who rang the bell the other morning sharply, and when the over-worked housemaid toiled up three pairs of stairs told her "the fire wants making up." The same nurse (?) thought that keeping the medicine glass clean and the patient's room dusted were trifles quite beneath her dignity, but possibly such behaviour is exceptional. If a nurse is "only a woman" after all, and not an angel, yet all of us know so many good women that naturally some can boast of acquaintance with a number of really good nurses.

### Our American Letter.

Amongst the recently established Alumnæ Associations news comes to us of one at St. Louis, Mass., formed by the graduates of the Protestant Hospital. The superintendent of nurses has accepted the office of president, and secretaries and treasurers have also been duly appointed. Medals are becoming quite a feature in American training schools, and the first class of graduates at the Lowell General Hospital, Mass., have just been presented with silver ones in addition to their "diplomas." At other hospitals either badges or medals accompany the certificates given to the graduates on the completion of their training, the less enduring gift of a bouquet of flowers being less popular. The post of head nurse at the Gouverneur Hospital has been conferred upon Miss Cassady, who received her training at the New York City Hospital. At Minneapolis a "Trained Nurses' Association" has been recently formed for mutual improvement and social intercourse between the nurses of that city.

Sincere regret has been expressed by all who know her at Miss Orr's resignation of the post of superintendent of the Paterson General Hospital, where her excellent work during the last eight years has endeared her to a large number of pupils and other friends.

At Newport, U.S.A., the district nurse has for a long time past made use of a bicycle in her daily rounds of visits and finds the saving of time and strength most satisfactory.

During the recent visit to Europe of Dr. and Mrs. Hunter Robb (Miss Isabel Hampton) the latter passed a few days in London in company with her friend and successor in office at the Johns Hopkins Hospital. These ladies gladly took the opportunity of renewing their acquaintance with the methods of teaching favoured by the London training schools.

At the meeting of American superintendents next year it seems probable that the subject of a national organisation for nurses will be discussed. This will include the suggested pension fund, also a scheme for a directory, with or without registration, &c.

### Minor Appointments.

Chelsea Hospital for Women.—Miss Jessie Southwell has been made Night Superintendent to this hospital. She was trained at St. Bartholomew's Hospital, holds excellent testimonials, and has gained the diploma of the London Obstetrical Society.

### Everybody's Opinion.

[Correspondence on all subjects is invited, but we cannot in any way be responsible for the opinions expressed by our correspondents. No communications can be entertained if the name and address of the correspondent is not given, or unless one side of the paper only be written on.]

### "OUR PRINCESS'S" COLOURS.

"A POLICYHOLDER" writes: As a recipient of the Princess's badge I should be glad to know if it is intended to be worn at all times. Fearing that it will become soiled, I am inclined to keep mine for state occasions, but of course, I wish to do the same as other nurses. I should like to express my sense of gratitude for the very pleasant time spent on July 26th. I feel sure all the nurses present thoroughly enjoyed themselves, and will remember the occasion as a red-letter day in their existence. I, for one, came away from Marlborough House with a feeling of regret that such meetings were not more frequent. Nurses have so few oppportunities of meeting in large numbers, and it is so pleasant to gather together and see old friends with whom one has, perhaps, not had any intercourse for years. Clergymen and doctors have their annual meetings, why should not similar gatherings be organised for nurses?

### A NURSE'S FEE.

"A MONTHLY NURSE" writes: It is usual to have a date in writing from the lady when making an engagement. If the lady breaks this contract she generally sends to the nurse, and should she be unable to attend, she supplies a deputy until she can nurse the patient herself. To thoroughly ignore her, and write that a temporary one was in the house, shows that the engaged nurse was not studied at all. I should claim my full fee in such circumstances, and board wages for six weeks. A solicitor tells me I should be perfectly correct in doing this. Of course, if a lady is nice, and treats you as a "woman," then some arrangement of half-fee might be made.

### REDUCED FEES.

A PRIVATE NURSE writes: As one of those who cannot afford to join the Pension Fund I feel there must be many old private nurses who are like myself in this respect unless they have kept all their earnings for themselves. Working on my own account I have often taken a reduced fee and adapted myself to the patient's means when I knew them to be limited. The well-known surgeon for whom I worked often would give me a hint, and when I knew he would take no fees I felt I must make a reduction in my own. My full fee for the largest operations when working for him was a guinea and a half per week, and only since he gave up practice have I sometimes charged two guineas. In his time I worked gratuitously among the poor, and that meant spending a good part of what I had earned from rich patients. Sometimes they made me presents, which made it difficult to charge them the full fee. It is asked "how, without the help of the Pension Fund, provision can be made for old age, if so, in what way? If not, would it not be better to economise now?" Can anyone tell me in what way this can be done? I lead the most frugal life and help others, and have often tried to get some more certain work to aid me possibly to join the fund, but without success. I must now trust to Providence, hoping that some of the great surgeon's old patients and friends will help to pension his old nurse.

[Much as we regret the present position of our correspondent (shown by her own words), we cannot acquiesce in her suggestion that any precedent for the alteration of a nurse's fee exists in the fact of a successful surgeon operating without pay. We think few doctors would approve of such a procedure on the part of nurses. Probably the surgeon named by "A Private Nurse" was quite unaware of her pecuniary position, otherwise he could not reasonably have "given a hint" nor have countenanced her gratuitous work amongst the poor. Presents can never take the place of fees, and in many cases are better declined altogether. Our correspondent's letter is too long for insertion in its entirety, but it certainly forms a strong warning to nurses to make provision for their own future.—ED. *T. H.*]

### Care of the Sick in Alexandria and Cairo.

### VI.—THE FRENCH HOSPITAL.

THIS, founded in 1816, was one of the first hospitals in Alexandria. It is very large, and under the care of the Sisters of St. Vincent de Paul. It contains 200 beds, and is capable of taking a good many more patients than any other institution we had previously seen, and adds to its usefulness by a special dispensary for eye cases. A private room costs from six to eight francs a day. Sailors pay three francs, and ordinary people two. There are separate rooms for cases of infection. The work is done by a Sister Superior, sixteen other Sisters, and two helpers. Of the four doctors one is French, one Italian, and two are German.

The men's part of the hospital is quite separated from the women's. The offices are in the basement, and instead of ordinary doors to the rooms, net curtains are hung to prevent the entrance of flies.

The ophthalmic wards are painted green, and are usually quite full. One room is set aside for very old people, who looked very cosy, sunning themselves in the broad verandah outside. We were pleased to find a number of bath-rooms plentifully supplied with water, also good sanitary arrangements.

Dinner was being served in all the wards when we arrived, and it consisted of soup neatly dispensed by a Sister, and carried to the bedsides by her helpers. Each bed was surrounded by mosquito curtains, and as the windows were wide open on to the verandah we were charmed to see little birds flying fearlessly in and out, hopping along the floor, and balancing themselves on the corni ce, making the place cheerful with their twittering.

The private rooms had little ante-chambers attached, and the couches, arm-chairs, and pictures made them look quite home-like. The hospital is managed by a committee, which unfortunately issues no printed report; merely having one written for private use. The kitchen is excellent, large and airy, with wire doors and a good stove, while the food is of the best quality and well prepared. The pharmacy, under the care of one of the Sisters, is especially complete, and consists of three rooms, one devoted to the washing of bottles, &c., and the other two to storing preparations and distribution of medicines. The hospital covers a good deal of ground, and consists of many storeys; corridors and staircases are all spotless, and not the faintest smell anywhere.

There are four houses in the city for out-patients. The Sisters are bright, gentle, cheery, and kind, but some of them are too old for the heavy and incessant work.

### Notes and Queries.

#### Queries.

(227) *Noma's Paste.*—Can you tell me what this is composed of, and what it is used for?—*Puzzled Pro.*

(228) *District Nurse.*—Is £50 per annum, with £5 for uniform, adequate remuneration for a fully-trained district nurse?—*Inquirer.*

#### Answers.

(227) *Noma's Paste (Puzzled Pro).*—The paste is composed of oxide of zinc five parts, glycerine eight parts, water six parts, gelatine six parts. The gelatine is first dissolved in the water, then everything is well mixed together while it is hot. In applying this as a splint no dometic bandage is necessary; only several layers of carbolised gauze bandages, over which the paste is painted with a brush while hot. If there is a wound no paste should be applied over it, and then the gauze bandage can be cut away to allow of the application of dressings. This is done without disturbing the splint which has been formed by means of Noma's Paste.

(228) *District Nurse (Inquirer).*—You do not say whether this salary is exclusive of full board and lodging, as it ought certainly to be.

### Jottings from Murree.

LADY ROBERTS' NURSES.

Murree is situated upon one of the southern slopes of the Himalayan Mountains; it occupies the upper end of one of those long and lofty spurs which are numerous on the Indian side of the Himalayas; its height is not uniform, but consists of several portions rising like steps one above another. The general elevation varies about 7,000 to 8,000 ft. The sides of the hills are more or less thickly covered with forest trees. A great variety of the ordinary English flowering plants may be found, such as honeysuckle, roses, clematis, and jessamine, and in the spring quantities of sweet-smelling violets and maidenhair and other ferns in perfection.

Murree is about forty miles from Rawalpindi, and the journey can be performed in five hours by Hill Tonga. The road passes over a lovely undulating country, beautifully wooded. Murree was first used as a sanatorium in 1851, shortly after the annexation of the Punjab. It is now the headquarters of the Punjab Army and Frontier Force. The chief buildings are the church, post office, kutchery, the station hospital for British troops, and the Lady Roberts Hospital for sick officers. Perhaps it may interest some of the readers of The Hospital to know something of the work carried on in the last-named hospital. Here an officer is able to obtain skilled nursing care which is so necessary to the preservation of life in sickness, combined with home comfort, both of which are so difficult to obtain in this country. Each officer has a separate ward, and during times of convalescence there is an officers' mess-room where they can read and write and make themselves generally comfortable. Of the eighty or ninety officers who have been admitted, most of them were suffering with enteric fever, generally young fellows fresh from England, who seem more ready to catch any enteric germs that may be hovering about, than the older men. This season three officers belonging to the Chitral Expedition, two of whom were wounded in the Malakund Pass, were transferred from the base hospital of the relief force to Murree, and the change was of great benefit to the officers, who speedily recovered from their wounds. The hospital is under the superintendence of the medical officer in charge of the station, and no patient can be admitted without his sanction. There are two nursing sisters who are assisted in their work by soldier orderlies. There is also a lady housekeeper whose work it is to look after the servants and general management of the house. All the officers speak in terms of high praise of the care and attention and home comforts which they receive. The nursing sisters not only work in this hospital but in the station hospitals during the winter months, and in times of emergency are sent to nurse sick officers in the plains or wherever they are required. There are few stations in the Punjab which have not, at some time or other, received the help of one or other of Lady Roberts' nursing sisters, of whom it has been truly said, that

"They are ever ready and ever true
To the toils and tasks they have to do."

### Appointments.

[It is requested that successful candidates will send a copy of their applications and testimonials, with date of election, to The Editor, The Lodge, Porchester Square, W.]

West Ham Infectious Diseases Hospital.—Miss Minnie Drakard has been appointed Matron of this hospital. She was trained at Beston Hospital, where she was afterwards assistant and head nurse. Miss Drakard held the post of staff nurse and sister of fever house at Nottingham General Hospital, and of matron at Taunton Sanitary Hospital. We wish her success in her new work.

Manchester Hospital for Consumption and Diseases of the Throat.—Miss Thompson, whose appointment as Matron of this hospital was inserted last week, was at one time matron of Brixham Cottage Hospital. Miss Thompson was trained at the Western Hospital, Glasgow, also 2s. 6d. kindly sent by sister Molra, this week.

### For Reading to the Sick.

GOD'S CARE.

**Motto.**

God hath promised to keep His people, and He will keep His promise.—*Anon.*

**Verses.**

There is no place where earth's sorrows
Are more felt than up in heaven;
There is no place where earth's failings
Have such kindly judgment given. —*Faber.*

Be still my heart! These anxious cares
To thee are burdens, thorns, and snares;
They cast dishonour on the Lord,
And contradict His gracious word.

Brought safely by His hand thus far,
Why wilt thou now give place to fear?
How canst thou want if He provide,
Or lose thy way with such a guide?

Did ever trouble yet befall,
And He refuse to hear thy call?
And has He not His promise passed,
That thou shalt overcome at last?

Though rough and thorny be the road,
It leads thee home apace to God:
Then count thy present trials small,
For heaven will make amends for all!
—*Hymns of C. and F.*

But Thou art true Incarnate Lord,
Who didst vouchsafe for man to die;
Thy smile is sure, Thy plighted word
No change can falsify. —*Wordsworth.*

Leaving the final issue in His hands
Whose goodness knows no change, Whose love is sure,
Who sees, foresees, Who cannot judge amiss.
—*Wordsworth.*

**Reading.**

Hagar was only a poor outcast, a hopeless woman wandering in the desert. Weary with aimless travel, with food and water exhausted, and with a great ache in her heart for the innocent boy by her side, she yields to the terrible pressure upon body and mind, and only waits for death to relieve them both of their troubles. "What aileth thee, Hagar?" Oh, the comfort of a sympathetic word when one's heart is breaking! And she had heard that voice before. It was the voice of a heavenly messenger sent of God to help her. Then the sorrowing ones of earth are known in heaven. Her name is spoken by the angel, even the name of this outcast bondwoman. There is One Who "carries our sorrows," Who takes our burdens as His own. "The very hairs of your head are all numbered." Our heavenly Father is concerned about knowing the minutest interests of our life. Yet how good it is just to tell our troubles to a sympathetic hearer. He knows, nevertheless; He listens to our story of trial and need; He invites our confidence. The things we cannot tell to others, the things our dearest earthly friends could neither understand nor rightly judge we may unfold to God without reserve. . . . "He is able to supply all your needs." "He is able to do exceeding abundantly above all that we ask or think." These are God's promises to the sad and sorrowing. "I will even make a way in the wilderness, and rivers in the desert. I will bring the blind by a way they knew not; I will make darkness light before them, and crooked things straight. These things will I do unto them, and not forsake thee."—"*The Life of Faith.*"

